# Novel Methods for Surface EMG Analysis and Exploration Based on Multi-Modal Gaussian Mixture Models

**DOI:** 10.1371/journal.pone.0157239

**Published:** 2016-06-30

**Authors:** Anna Magdalena Vögele, Rebeka R. Zsoldos, Björn Krüger, Theresia Licka

**Affiliations:** 1 Multimedia, Simulation and Virtual Reality Group, Institute of Computer Science II, University of Bonn, Bonn, Germany; 2 Working Group Animal Breeding, Department of Sustainable Agricultural Systems, University of Natural Resources and Life Sciences Vienna, Vienna, Austria; 3 Gokhale Method Institute, Stanford, CA, United States of America; 4 Movement Science Group, Department for Small Animals and Horses, University of Veterinary Medicine Vienna, Vienna, Austria; 5 Royal (Dick) School of Veterinary Studies, University of Edinburgh, Edinburgh, Scotland, United Kingdom; Semmelweis University, HUNGARY

## Abstract

This paper introduces a new method for data analysis of animal muscle activation during locomotion. It is based on fitting Gaussian mixture models (GMMs) to surface EMG data (sEMG). This approach enables researchers/users to isolate parts of the overall muscle activation within locomotion EMG data. Furthermore, it provides new opportunities for analysis and exploration of sEMG data by using the resulting Gaussian modes as atomic building blocks for a hierarchical clustering. In our experiments, composite peak models representing the general activation pattern per sensor location (one sensor on the long back muscle, three sensors on the gluteus muscle on each body side) were identified per individual for all 14 horses during walk and trot in the present study. Hereby we show the applicability of the method to identify composite peak models, which describe activation of different muscles throughout cycles of locomotion.

## Introduction

Over the last decade, monitoring and analysis of muscle activity during locomotion has gained increasing popularity as novel wireless technology has made recording of such data much easier. The different surface electromyography (sEMG) signal capturing techniques and processing methods are controversially discussed [1]. All processing methods have advantages and disadvantages as they affect the sEMG signal. While they are reducing noise, some information content may also be lost either in the process of smoothing the linear envelope, or by normalization of the time scale and amplitude [1]. Therefore, researchers have focused on optimized processing methods—e.g. EMG rectification as a necessary pre-processing step (Farina et al. [2], Negro et al. [3]). In earlier sEMG studies, single events such as maximum activity of filtered traces were reported. However, in recent years additional, more complex information is commonly obtained, such as motor modules extracted from the EMG signals (Gizzi et al. [4]). Besides the analysis of general characteristics of sEMG signals during full trials of locomotion, evaluation of sEMG peaks during individual cycles of motion yields valuable insight. Additionally, muscle activation patterns prior to and after peak activation are of special interest, as they could generate more compact models of information in relation to the movement pattern as well.

In human and animal biomechanics, there are three applications which dominate the use of the sEMG signal: its use as an indicator for the initiation of muscle activation (a question of movement pattern), its relationship to the force produced by a muscle (a kinetic question), and its use as an index of the fatigue processes occurring within a muscle (a muscle physiology question) [5].

To achieve these goals in the field of animal biomechanics, a number of different data processing techniques are currently employed, this is incompatible with the direct comparison of the results of different studies (Boudaoud et al. [6], Olsen et al. [7] Williams et al. [8]). A standard for data processing which is already existing in human biomechanics [9] would therefore be beneficial in sEMG studies in animals, even if it is in addition to individual data processing techniques employed for specific questions.

Interpretation of sEMG in dynamic contractions has its own difficulties even in humans (cf Farina [10]). Maximally voluntary contraction (MVC) is commonly used in humans as sEMG reference value, however, this is not possible in animals, but is also sometimes difficult or even impossible to obtain in humans, e.g. during swimming (Martens et al. [11]). Also, it is unclear whether the MVC of a specific muscle is actually representative for e. g. the use of the same muscle during locomotion [12].

Of all animals, horses have most commonly been investigated using sEMG in a large number of studies (cf Valentin and Zsoldos [13]). This is due to the importance of their musculoskeletal system for their use, but also due to the large superficial muscle areas available for sEMG such as leg and back muscles. Early on, studies on the long back muscle were carried out at stance with volitional movements (Peham et al. [14]) as well as during locomotion (Licka et al. [15, 16]).

Overall, it is still more common to have measurements carried out during locomotion, for example comparing muscle patterns between gaits. In horses, different back and leg muscles are often investigated to show, analyse and interpret homogenous cycle patterns during dynamic conditions. The m. longissimus dorsi (long back muscle) is one of the most commonly investigated equine muscles (E. g. Cottriall et al. [17], Licka et al. [16]). This large surface muscle of the back is an ideal candidate for the investigation of spinal stabilization due to its function of extending the back (during bilateral contraction) and splinting the back against passive deformation (during uni- and bilateral contraction). Besides trunk muscles, limb muscles are also often investigated (cf. Zaneb et al. [18], Crook et al. [19, 20], Williams et al. [21], as they directly influence efficient locomotion which is an important area of research.

The advantage of studying the sEMG of large muscles is that they control larger movements requiring greater strength, and they may contain 100 to 1000 fibres per motor unit (Rash and Quesada, [22]). Up to now, most studies have used only single sEMG electrodes when investigating large superficial leg muscles of the horse (Williams et al. [21]; Zaneb et al. [18]; Wakeling [23, 24]). As the cone of muscle activity measured underneath an sEMG electrode can only sample those motor units with muscle fibres located within 10–12 mm of the electrodes and thus contributing significantly to signal energy (Fuglevand et al. [25]). This approach has obvious drawbacks. Array sEMG, a multi-electrode grid sampling method, on the other hand is capable of recording motor unit potentials to estimate muscle fibre conduction velocity (Zwarts et al. [26]). For the use in differentiating the activity in different parts of a large muscle these electrodes are too close to each other and it is therefore not expected to replace electrodes in this regard.

In the present study, leg muscle activation at three different locations will be investigated, because muscle fibre types have been shown to vary between the different sections of the equine gluteus medius muscle (as was described histologically by Bruce [27] in the m. gluteus medius of Thoroughbreds). A similar intra-muscular pattern was previously identified in the m. longissimus dorsi where activity was measured at three different positions on the same muscle (cf Licka et al. [15, 16]), showing the time line of the activation of this long muscle.

Empirical mode-seeking or fitting of Gaussian mixture models (GMMs) has never been applied in the investigation of biomechanics of animal movements up to now, despite it being advantageous for a variety of fields of research (Mazoyer et al. and Spainhour et al. [28, 29]). Fitting GMMs has been used in signal processing and pattern recognition (cf Bishop [30]). In particular, the method can be applied to classification-related problems as in human action recognition based on sEMG data (cf. Ju et al. [31], Ding et al. [32]). In heterogeneous data such as sEMG there is the need to explore data by hierarchical clustering, which helps to restructure the data (cf Bernard et al. [33], Wilhelm et al. [34]). Especially, clustering complete multivariate time series data (Rodrigues et al. [35], Rani and Sikka [36]) helps discover relations between the complete group and subgroups of individuals with respect to average, typicality and anomaly. These methods have the potential to detect subgroups based on sEMG data, and they are therefore employed in the present study. While the application of different statistical methods exists for the investigation of animal muscle activity during locomotion, the value of detection of clustered peaks in sEMG signals and estimation of GMMs to describe these data has not yet been documented.

Therefore, the purpose of this study was to characterize with a new exploratory method the equine trunk and limb muscle activation during different gaits, and, more specifically, (a) to document the variability of muscle activation at the three locations of the most studied muscle of this study (gluteus medius); (b) to identify the structure of composite peaks within cycles of muscle activity (gait cycles of locomotion); and (c) to identify the gait dependency of these muscle activity patterns.

## Materials and Methods

### Ethics statement

Experiments were carried out under the University of Veterinary Medicine Vienna’s animal experiment licence for the horses of the university teaching herd. All experiments and also this particular study were approved by the institutional ethics committee, the Advisory Committee for the scientific use of live animals (Ethik-und Tierschutzkommission, ETK) of the Vienna University of Veterinary Medicine, and the national authority according to § 8ff of Law for Animal Experiments (Tierversuchsgesetz TVG, bmwf GZ 68.205/0160/*II*/3*b*/2012), more specifically the part covering non-invasive orthopaedic examinations and measurements.

### Horses

Fourteen horses without clinical sign of back pain or lameness were used in this study (14 Haflinger mares, mean age was 8 ± 3 years, CI (6, 9), range 4–14 years; mean body mass was 463 ± 42 kg, CI (439, 487), range 396–526 kg; mean height at the withers was 131 ± 5 cm, CI (128, 134), range 125–145 cm.

Two larger muscles were investigated, the m. longissimus dorsi (trunk muscle) and the m. gluteus medius (leg muscle). Muscle activation was recorded by sEMG at one location on the m longissimus dorsi and at three different locations of the m. gluteus medius.

#### Longissimus Dorsi

The equine m. longissimus dorsi is the longest major back muscle of the horse, which main function is spinal stabilization. At the level of the 16th thoracic vertebra, where the maximal lateral movement of the spine occurs in walk and trot—sEMG electrodes were placed parallel to the muscle fibre direction—over both the left the left (LDL) and right (LDR) m. longissimus dorsi (cf. Licka et al. [15] Wakeling et al. [24] Groesel et al. [37]).

#### Gluteus Medius

The equine m. gluteus medius (GM) is the largest unsegmented muscle of the horse. Its main function is to extend the hip and retract and abduct the limb. The muscle provides the visible shape of the croup, and due to its location directly under the skin, it is an ideal candidate for detecting intra-muscular activation differences with sEMG.

#### Electrode Placement

The following procedure was applied before electrode placement, skin was shaved and the resistance was reduced by thoroughly cleaning the shaved skin with slightly abrasive, roughly woven swabs. Surface electromyography activities were collected with wireless electrodes (each sensor consisting four parallel silver bars with an integrated amplifier, size 27 x 37 x 15 mm, mass 14.7 g, CMMR > 80 db, Baseline noise < 750 nV RMS) placed bilaterally over the left and right gluteus medius muscle at roughly the midpoint between origin and insertion about 5cm apart on the lateral (GM1), middle (GM2), and medial (GM3) part of the gluteus medius muscle and over the left and right longissimus dorsi (LD) muscle at the level of 16th thoracic vertebra approximately 5 cm lateral to the dorsal spinous process. These electrodes were positioned parallel to muscle fibre orientation and were fixed to muscles using the Delsys Adhesive Sensor Interface™.

### Data Acquisition

For this study, a set of three-dimensional kinematic data and surface electromyography (sEMG) in walk and trot were synchronously collected.

#### EMG

Surface electromyography measurements were taken in the above mentioned 14 horses without lameness, walking and trotting on a treadmill. Measurements were carried out by a set of 8 trunk-mounted sEMG sensors of a Delsys Trigno Wireless System (Boston, MA, USA). The resulting EMG signal was full-wave rectified and sampling rate was reduced to 120 Hz. A Butterworth low-pass filter was applied (fourth order; cut-off frequency, 20 Hz). For each horse three trials or more were captured per gait each of them 10 seconds in walk and in trot.

Horses were exercised on a treadmill performing two different gaits, walk and trot. Each subject was recorded over a period of 10 seconds per gait, repeating each trial run 3 times in a row. The recordings resulted in one data file per trial run. The frame rate of the recordings was 4000 frames per second.

The study followed the guidelines laid out in the works of de Luca [5], Hermens [9], and Merletti [38]. Note that there is not a standard work on guidelines for equine (or any animal) sEMG data processing and reporting so far.

#### Kinematic Data

During each trial, data was captured simultaneously using a synchronized setup of the recording systems for kinematic and EMG data. The motion of the left fore was recorded by optical Motion Capture (10 high speed cameras; Eagle Digital Real Time System, Motion Analysis Corp., Santa Rosa, CA, USA) simultaneously to the sEMG recordings. Since the two systems were recording simultaneously by design, the kinematic data of the left fore reflect the same motion patterns as the EMG data.

For kinematic measurements seven reflective skin markers were positioned on each horse using adhesive tape; one on the forehead, one on the highest point of the withers, on the sacrum were placed and on the lateral side of each hoof to identify motion cycles. Three-dimensional kinematic data in walk and trot were collected using ten high-speed cameras recording at 120 Hz.

### Data Processing

#### EMG Data

Consider a data signal *M* in the set of recorded data describing activation of one muscle. *M* is a discrete function *M* = (*f*_1_, …, *f*_*n*_) of length n∈N in time where each *f*_*i*_, 1 ≤ *i* ≤ *n* encodes the amount of muscle activation at time stamp *i*.

The EMG signal is rectified (full wave rectification) in the sense that

$$M_{\text{rect}}=\left(|f_{1}\left|,\ldots ,\right|f_{n}|\right).$$ (1)

The sampling rate of the raw data sequence was reduced to to 120 Hz by a factor of 120/4000 = 0.03 while at the same time applying a lowpass filter to prevent aliasing.

This pre-processing step was completed by filtering by a fourth order Butterworth low-pass filter (cut-off frequency: 20 Hz) in order to reduce noise.

#### Kinematic data

kinematic data are cleaned and interpolated to remove temporal gaps in order to keep track of the movement of the left fore limb.

Three-dimensional coordinates of each marker during the time course of each experiment were calculated from the data using kinematic software. These time series were then filtered by a Butterworth low-pass filtered (cut-off frequency, 10 Hz). The data was split to motion cycles starting with the stance phase of the left fore automatically. We also calculated characteristics of each motion cycle like its duration and range of values. Instead using automatic motion segmentation techniques [39, 40], we use stance phase information to be consistent with previous work in the biomechanics domain.

#### Detection of Cycles in Kinematics and EMG Data

**Fig 1:**
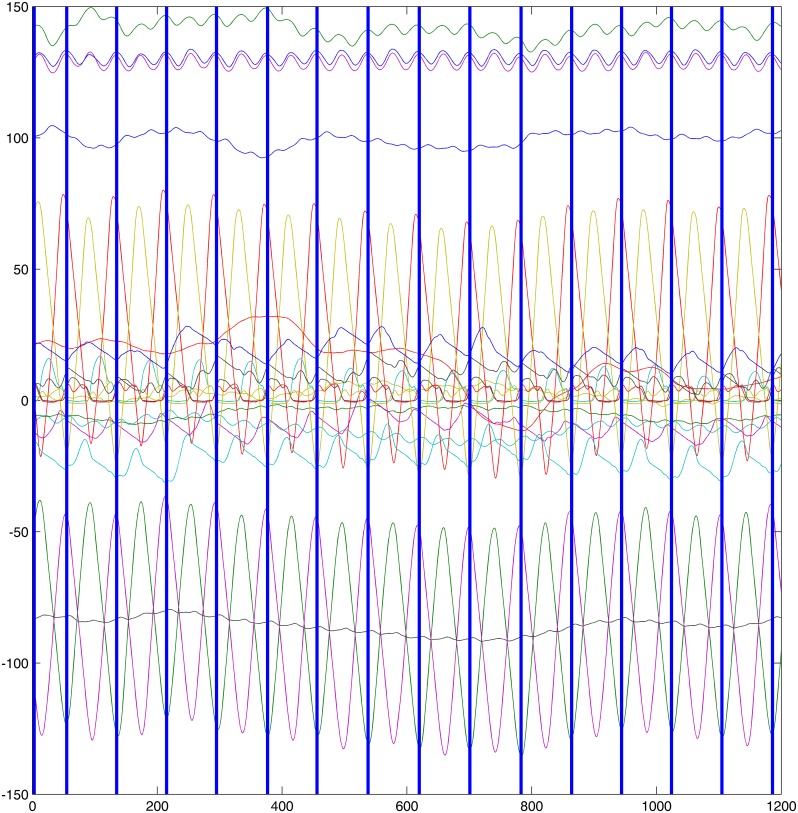
Kinematics information trot. Left fore. The orange line illustrates the motion in the y-axis, i. e. the ground contacts occur at the beginning of flatter episodes of this orange curve.

**Fig 2:**
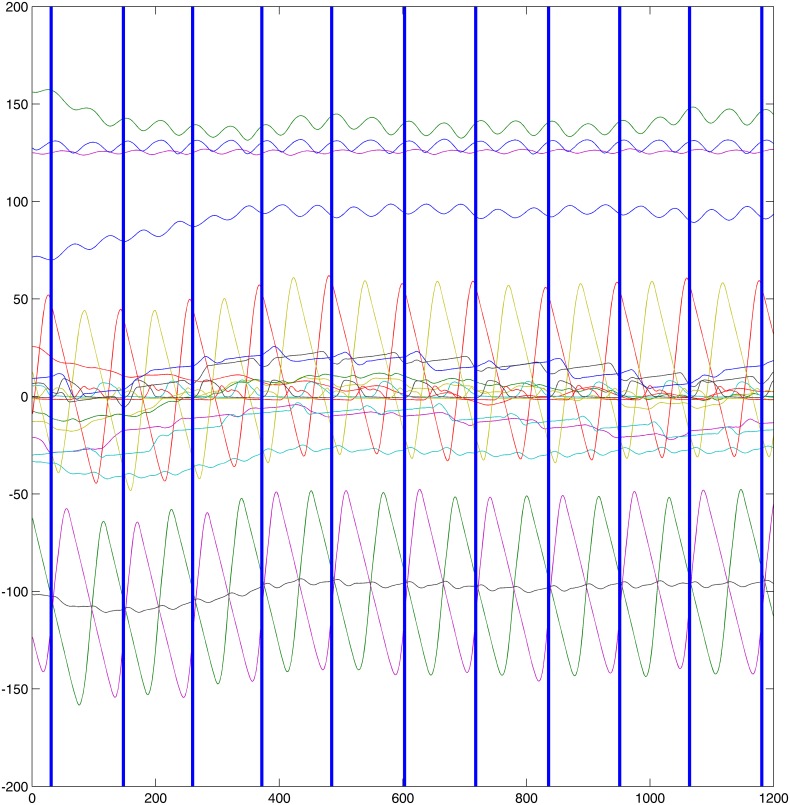
Kinematics information walk. Left fore. The orange line illustrates the motion in the y-axis, i. e. the ground contacts occur at the beginning of flatter episodes of this orange curve.

The kinematics information on the left fore limb is used for ground contact detection (see Figs 1 and 2). This detection of ground contacts results yields estimations of stance phases of the associated hoof and thus in the detection of motion cycles. Each cycle of motion includes the sequential ground contacts of all four hooves starting with the contact of the left fore. Note that it is custom to define the notion of footfall patterns consistently starting with the contact of one particular foot (refer to Hill [41] or Robilliard [42] for further readings). Since the two data sets were recorded simultaneously, the segmentation of kinematic data into cycles can be directly transferred to the EMG data. This results in a disjoint separation of the signal *M*

$$M_{f}=\left(s_{1},\ldots ,s_{m}\right)$$ (2)

where $s_{j}=(f_{j1},\ldots ,f_{j\lambda }),\lambda \in N$ for 1 ≤ *j* ≤ *m*. Each *s*_*j*_, 1 < *j* < *m*, represents one complete cycle of the recorded gait. Note that *s*_1_ and *s*_*m*_ may be incomplete cycles due to being at the beginning, respectively at the end. Refer to Fig 1 for an example (compare to gait event detection of Olsen et al. [7]).

#### Normalization

All EMG signals per cycle were normalized in the time domain as well as in the amplitude domain. All cycles had different phase timing both within and between subjects, therefore each cycle was mapped to a 100 point scale to allow for comparison between cycles in the time domain (cf Martens et al. [11]). Each cycle was also scaled to the interval [0, 1] ([11], [15]).

### Fitting of Gaussian Mixture Models

Consider a set of cycles $S=\{s_{1},\ldots ,s_{K}\}$ belonging to the same class of trials (trot vs. walk). Let (*p*_*j*_1__, …, *p*_*j*_*l*__) denote the set of all local maximal in cycle *s*_*j*_. Let *S* denote the set of all maxima for all 1 ≤ *j* ≤ *K*. We hypothesize that the EMG peaks in this set *S* can be modeled by a Gaussian mixture distribution

$$P\left(x\right)=\sum_{i=1}^{n}w_{i}N\left(\mu_{i},\Sigma_{i}\right),$$ (3)

i.e. the peak data are distributed according to a two-dimensional multi-modal density function

$$f\left(x;\mu_{1},\ldots ,\mu_{n},\Sigma_{1},\ldots ,\Sigma_{n}\right)=\sum_{i=1}^{n}w_{i}\left(p\left(x;\mu_{i},\Sigma_{i}\right)\right)$$ (4)

where *μ*_*j*_ = (*μ*_*j*1_, *μ*_*j*2_) is the two-dimensional mean point and *Σ*_*j*_ is the covariance matrix of the *j*th distribution. In more detail, each of these distributions is of the form

$$p\left(x,y\right)=\frac{1}{2\pi \sigma^{2}}e^{\frac{{\left(x-\mu_{1}\right)}^{2}+{\left(y-\mu_{2}\right)}^{2}}{2\sigma^{2}}}.$$ (5)

In order to fit a suitable GMM model to a given set of peak points *S*, we first need to determine the number of components needed to represent this model. The other parameters, i. e. model means *μ*_*i*_, covariances *Σ*_*i*_ and the weights *w*_*i*_ for each 1 ≤ *i* ≤ *n* are then estimated by the expectation maximization (EM) algorithm (cf. Bishop [30]). Refer to the methods section for details.

One particular difficulty is we do not know how many components can model the data set best. However, this number is crucial for initialization of the GMM. There have been a number of works that discuss initialization of EM algorithms on a more general level. As Blömer and Bujna discuss against the backdrop of theoretical computer science [43], the EM algorithm is often sensitive to the choice of the initial parameter vector. The same has also been observed by Melnykov and Melnykov [44] especially in settings where the number of mixing components is unknown.

Therefore, it becomes clear that suitable initialization needs to be found in the present case. Based on this observation and the findings of the more general works [45, 46], the problem of finding an initialization vector for the EM algorithm is solved by a clustering approach. The mean sEMG signal per sensor, gait and subject was found to be a robust estimation of the individual signals per sensor, gait and cycle. Therefore, all peak data of one subject and gait and sensor are assigned to multiple clusters where each individual cluster yields initialization estimations for one mode in the multi-modal Gaussian distribution. The number of clusters is determined by finding the peaks of a mean curve as outlined below.

#### Clustering Peak Values

The first step is to robustly estimate a parameter k∈N approximating the number of peaks of a given collection curves representing cycles. Since computation of all maxima per cycle may result in a different number for each given cycle. Since the parameter should be a robustly estimated for complete sets of cycles of one class, a mean curve is computed as a reference and its peak values are computed as a representative of all cycles. Details on how the general algorithm employed to compute the peaks of a discrete function can be found in Mariscotti’s works on the subject [47].

The second step is then a temporal pre-clustering of peak locations (position of frame in the time dimension) by *k*-means algorithm resulting in a set of *k* disjoint clusters {*C*_1_, …, *C*_*k*_} for k∈N.

The *k*-means algorithm (cf Bishop [30]) is a standard method of partitioning points into exactly *k* clusters by minimization of the total intra-cluster variance

$$J=\sum_{j=1}^{k}\sum_{i=1}^{m}d\left(x_{i}^{\left(j\right)},c_{j}\right)$$ (6)

where *c*_*j*_ denotes the center of cluster *C*_*j*_ and the distance function is the squared distance of two points in the time dimension:

$$d\left(x,c\right)\;=\;∥x-c{∥}^{2}.$$ (7)

#### Finding Modes

Consider the set of all observations *C* = {*p*_1_1__, …, *p*_*k*_*c*__}, i. e. the full set of EMG peaks in all given cycles. Each point in *C* is a point in the space [0,…,100]×[0,1]⊂Z×R and the data set *C* can be represented by a *N* × 2 matrix *X* in which the *i*th row is given by the *i*th peak point and where *N* is the total number of peaks present in the data. A bivariate GMM describing *C* in the sense of Eq 5 can be fitted to the collection of points over all clusters by maximization of the log likelihood function. The log likelihood function is given by

$$\ln p\left(X|w,\mu ,\Sigma \right)=\sum_{i=1}^{N}\ln\left\{\sum_{j=1}^{K}w_{j}N\left(x_{i}|\mu_{i},\Sigma_{i}\right)\right\}.$$ (8)

where *p*(*X*|*Y*) denotes the conditional probability of *X* given *Y*. It is a well-known fact that there is no closed-form solution for this optimization problem if the number of components *K* is greated than 1 (cf Bishop [30]). The function ln *p*(*X*, *w*, *μ*, *Σ*) was maximized with respect to the mixing proportions *w*_*i*_ such that $\sum_{i=1}^{N}w_{i}=1$. This maximization of likelihood problem can be solved by a simple case of an iterative method called expectation maximization algorithm. This special case of the EM algorithm iterates two steps, the *expectation step* and the *maximization step*. In the expectation step, the posterior probabilities of Eq 8 are used given the current parameter estimation. In the maximization step, the probabilities resulting from the computation in the expectation step are used to update the estimation of the parameter vectors *w*, *μ* and *Σ*. Technical details on the procedure can be found in [30].

### Exploring Data by Hierarchical Clustering

Exloring data for subgroups that can be distinguished by patterns in the muscle activation information will deliver new insight. Hierarchical clustering allows for exploring a set of given data *D* in terms of such subgroups in an unsupervised manner. This is especially convenient when the number of subgroups is unknown.

**Fig 3:**
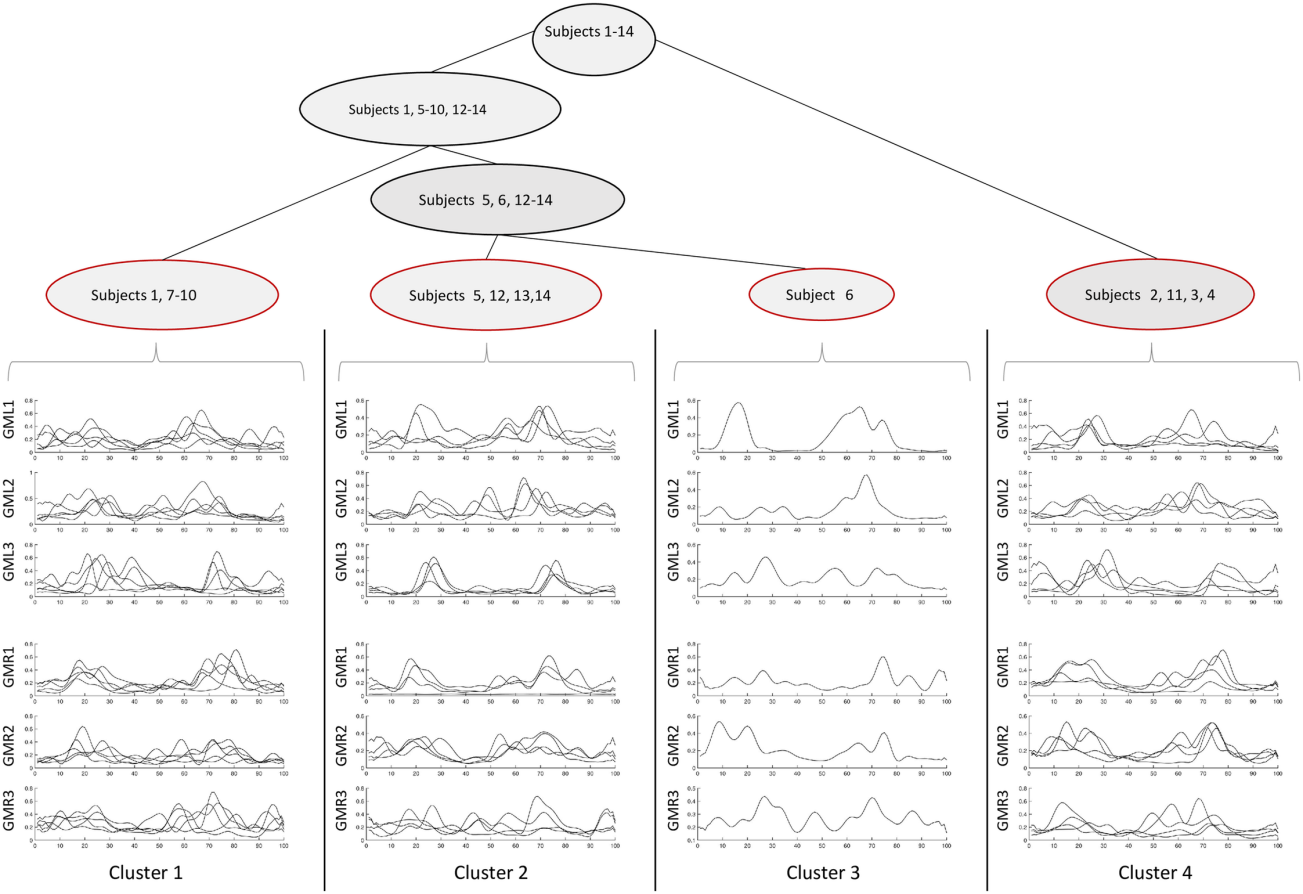
Cluster tree visualization, m. gluteus medius. Three sensors per side capturing trot. Note that only the upper part of the tree is shown, namely at a level where 4 clusters are created (red highlighting).

**Fig 4:**
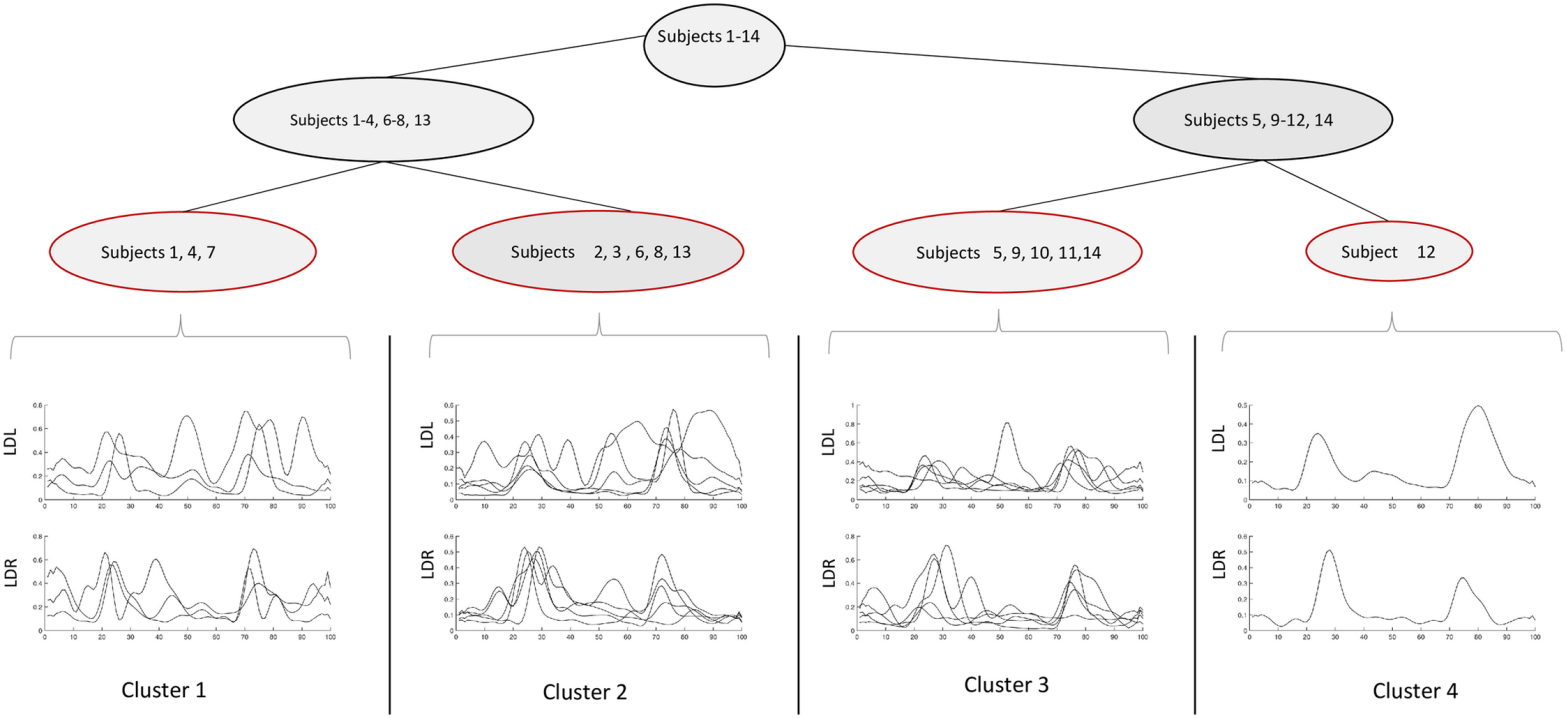
Cluster tree visualization, m. longissimus dorsi. One sensor per side capturing trot. Note that only the upper part of the tree is shown, namely at a level where 4 clusters are created (red highlighting).

Agglomerative clustering is achieved by iteratively carrying out three steps (cf Hastie et al. [48], Olson [49]). The first is step assigns each element of the data set its own cluster, the second step merges the two clusters which have the lowest distance. In a third step, the distances between the newly merged cluster and each remaining cluster is computed. Note that a number of different distance measures can be employed for the computation of cluster proximity in step two. Also, the third step depends on the measure employed to determine this distance, the so-called *linkage*. The three steps are repeated until all clusters have been merged to form one single cluster, that is, after *n* steps where |*D*| = *n*. Each iteration yields a partition of the full data set. The partitions of each iteration can be represented by a binary tree (see results in Figs 3 and 4). In the hierarchical structure of the tree, the different stages of the clustering are reflected as they develop from very fine-grained to coarser partitions (bottom-up).

### Measures of Variability

#### Mean Absolute Deviation

The intra-subject variability is given per sensor *S* as the cumulative Euclidean distance between the mean curve of all cycles in the signals to all individual cycles.

$$v_{S}=\sqrt{\sum_{i=1}^{l_{S}}{\left|\hat{c}-c_{S_{i}}\right|}^{2}}$$ (9)

where *l*_*S*_ denotes the number of cycles present per sensor. The mean absolute deviation (MAD) is given by

$$MAD_{S}=\frac{1}{n}\sum_{i=1}^{l_{S}}\left|\hat{c}-c_{S_{i}}\right|.$$ (10)

Note that the space in which the deviations are given is the space in which the curves live (remember there was a scaling to [0,…,100]×[0,1]⊂Z×R).

The inter-subject variability is given analogously to Formulas 9 and 10 for the mean curves of all individual per sensor.

#### Variance to Mean Ratio

Martens et al. [11, 50] introduce different measures of variability in their studies on muscle activation per cycle. The former is focused on swimmers, while the latter investigates walking patterns in children. The variance to mean ratio of a data set *X* of m∈N cycles is defined as:

$$CV=\frac{\sqrt{\frac{1}{n}\sum_{i=1}^{n}\sigma_{i}^{2}}}{\frac{1}{n}\sum_{i=1}^{n}\left|\bar{X_{i}}\right|}$$ (11)

where *n* is the number of frames per cycle and *σ*_*i*_ denotes the standard deviation at frame *i*.

The coefficient of variation can also be given per time frame *i* as

$$CV_{i}=\frac{\sigma_{i}}{X_{i}}$$ (12)

The term mean CV will refer to the mean of this time series.

#### Variability Ratio

The variability ratio of a data set *X* is defined as

$$VR=\frac{\frac{1}{n(m-1)}\sum_{i=1}^{n}\sum_{j=1}^{m}{\left(X_{ij}-\bar{X_{i}}\right)}^{2}}{\frac{1}{\left(nm-1\right)}\sum_{i=1}^{n}\sum_{j=1}^{m}{\left(X_{ij}-\bar{X}\right)}^{2}}$$ (13)

where *X*_*ij*_ is the data entry at frame *i* and at cycle *j*, *n* is the number of frames per cycle and *m* is the number of cycles in the data set.

#### Inter-quartile Ratio

The inter-quartile ratio between the 25th quartile *Q*_1_ of the data set at frame *i* and 75th quartile *Q*_3_ of the data set at frame *i* is computed as a measure of variability over time:

$$CQV=\frac{Q_{3}-Q_{1}}{Q_{3}+Q_{1}}$$ (14)

Note that these variability measures can be used for intra-individual as well as inter-individual variability computations.

### Comparing Probability Distributions

The similarity of probability distributions can generally be assessed by a distance measure between the respective distribution functions. In the classical case, i. e. for uni-modal probability models, this distance measure is given by the *Kullback-Leibler distance*. Unfortunately, the definition of this distance measure does not translate to multi-modal distributions which are of the form described by Eq 4. However, there is a similar distance measure, the *Cauchy-Schwarz distance* which is based on the following idea (cf Kampa and Principe [51]).

Given two multi-modal Gaussian distributions *P* and *Q*, the Cauchy-Schwarz distance between them is defined by

$${\text{dist}}_{CS}\left(P,Q\right)=-\log\left(\frac{\int Q(x)P(x)dx}{\sqrt{\int Q{\left(x\right)}^{2}dx\int P{\left(x\right)}^{2}dx}}\right).$$ (15)

This is a symmetric measure for arbitrary probability distribution functions such that 0 ≤ dist_*CS*_ < ∞. In particular, this measure translates to the special case of multi-modal Gaussian distributions when the distributions accommodate the same number of modes.

## Results and Discussion

### Results of Cycle Detection

**Fig 5:**
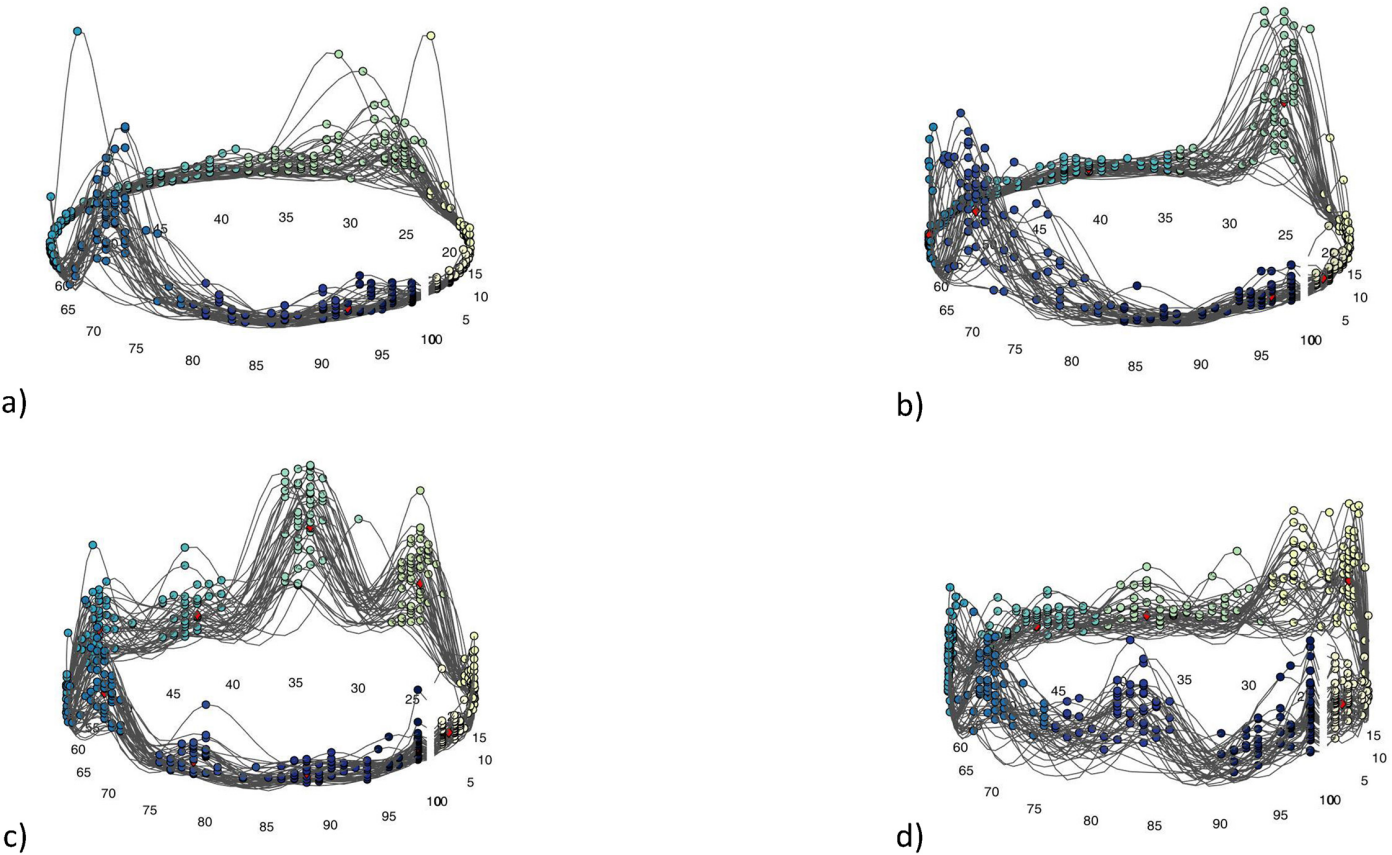
Cyclic structure of muscle activation. Example of one individual (subject 13) in trot. a) LDL, b) LDR, c) GML1, d) GMR1. Note the different muscle activation patterns across the two different muscles. The different colors of the data points refer to the different clusters initialized by peaks in the curves describing muscle activation throughout each cycle.

The data set investigated in the present study consisted of several trial runs per gait and subject. There are data of 48 trial runs (subject average: 3.43, mean absolute deviation: 0.55, total maximum 5, total minimum 3) of trot in total and 44 (subject average: 3.14, mean absolute deviation: 0.24, total maximum 4, total minimum 3) trials of walk. The trial runs were segmented by the procedure described in the section on EMG resulting in a total number of 709 motion cycles for trot and 426 cycles for walk. On average, each trial subject performed 51 (mean absolute deviation: 8.39) cycles of trot and 30 (mean absolute deviation: 3.18) cycles of walk. For an example visualization of the cyclic structure of muscle activation refer to Fig 5 Note that the duration of each trial was 10 seconds, so muscle fatigue is not a contributing factor. It can be observed that there is a smaller number of walk cycles than trot despite the fact that the total trial numbers do not significantly differ. This is explained by the difference in speed between the gaits.

#### Intra-Subject Variability of sEMG between Different Cycles

The mean curves used to initialize the fitting of GMMs tell us several things. On the one hand, they help determine how many consistently present peaks occur during one average gait cycle. On the other hand, the deviation of all cycles of the signal from this mean curve tell us how reliable this information is. The chosen measure of reliability is the intra-subject variability presented in Tables 1 and 2.

**Table 1:** Intra-Subject Deviations Trot.

Deviations from cycles to mean curve															
	Subject number	1	2	3	4	5	6	7	8	9	10	11	12	13	14
LDL	∅ deviation	0.93	1.04	1.22	1.08	0.94	0.67	1.52	1.49	1.32	1.87	1.0	1.02	0.71	1.04
	MAD	0.26	0.41	0.26	0.25	0.19	0.16	0.24	0.26	0.36	0.29	0.21	0.23	0.29	0.25
GML1	∅ deviation	1.22	1.25	1.22	1.11	0.64	1.33	0.77	1.15	1.21	1.18	1.58	1.22	1.03	1.12
	MAD	0.27	0.24	0.23	0.23	0.18	0.3	0.15	0.26	0.22	0.29	0.26	0.33	0.18	0.24
GML2	∅ deviation	1.18	1.13	0.78	1.21	1.12	0.97	1.29	1.15	1.11	0.99	0.84	1.0	1.4	0.89
	MAD	0.26	0.28	0.29	0.28	0.29	0.28	0.28	0.37	0.34	0.19	0.16	0.29	0.25	0.2
GML3	∅ deviation	1.05	1.15	1.1	1.48	0.17	1.19	1.08	1.09	0.98	1.09	1.18	1.22	1.12	1.06
	MAD	0.21	0.25	0.23	0.2	0.24	0.18	0.2	0.25	0.29	0.23	0.22	0.24	0.19	0.24
LDR	∅ deviation	1.18	0.94	1.03	1.26	0.81	1.0	1.1	0.94	1.24	1.09	1.04	0.71	1.07	0.01
	MAD	0.27	0.2	0.2	0.22	0.28	0.28	0.24	0.19	0.6	0.24	0.22	0.22	0.28	0.25
GMR1	∅ deviation	1.32	1.14	0.98	1.55	0.81	1.09	0.88	0.82	1.33	1.06	1.1	0.11	1.1	1.02
	MAD	0.29	0.28	0.3	0.3	0.16	0.2	0.25	0.2	0.25	0.21	0.26	0.16	0.18	0.26
GMR2	∅ deviation	1.31	1.14	0.97	1.12	1.06	1.05	1.05	1.3	1.32	0.97	1.11	1.12	1.54	0.91
	MAD	0.23	0.26	0.27	0.26	0.21	0.2	0.21	0.51	0.34	0.17	0.19	0.33	0.25	0.22
GMR3	∅ deviation	1.40	1.38	1.27	0.83	1.28	1.35	1.34	1.28	1.23	1.13	1.11	1.17	0.86	1.42
	MAD	0.21	0.32	0.36	0.3	0.22	0.24	0.24	0.3	0.23	0.18	0.18	0.39	0.2	0.23

**Table 2:** Intra-Subject Deviations Walk.

Deviations from cycles to mean curve															
	Subject number	1	2	3	4	5	6	7	8	9	10	11	12	13	14
LDL	∅ deviation	1.01	1.21	1.22	1.0	0.98	1.21	1.15	1.06	1.47	1.25	1.05	1.33	0.99	1.41
	MAD	0.24	0.19	0.25	0.19	0.22	0.19	0.24	0.25	0.41	0.22	0.27	0.19	0.34	0.28
GML1	∅ deviation	1.31	1.15	0.76	0.96	0.91	1.11	1.14	1.38	0.93	1.47	1.43	0.61	1.64	0.95
	MAD	0.29	0.2	0.22	0.14	0.18	0.28	0.24	0.21	0.37	0.25	0.26	0.36	0.24	0.2
GML2	∅ deviation	0.83	0.98	0.83	1.59	0.93	0.88	0.78	0.87	1.13	1.05	1.04	0.47	1.16	0.85
	MAD	0.25	0.19	0.25	0.3	0.19	0.22	0.16	0.28	0.24	0.22	0.2	0.35	0.16	0.34
GML3	∅ deviation	1.1	0.7	1.02	0.7	1.28	1.04	0.63	1.06	1.1	1.3	0.8	0.98	0.8	0.93
	MAD	0.25	0.14	0.19	0.18	0.17	0.2	0.17	0.18	0.22	0.25	0.22	0.37	0.22	0.39
LDR	∅ deviation	1.21	1.23	1.05	1.6	1.13	1.05	1.14	0.93	1.46	1.1	1.3	0.67	1.27	1.27
	MAD	0.34	0.36	0.25	0.23	0.21	0.23	0.25	0.19	0.21	0.2	0.27	0.23	0.23	0.22
GMR1	∅ deviation	1.05	1.27	1.09	1.14	1.14	1.41	1.23	0.94	1.32	0.97	1.07	0.1	1.24	1.07
	MAD	0.3	0.19	0.13	0.17	0.2	0.55	0.29	0.21	0.28	0.18	0.16	0.01	0.2	0.19
GMR2	∅ deviation	1.13	0.99	1.19	1.18	0.92	1.1	1.28	1.22	1.17	1.0	1.0	0.83	1.02	0.89
	MAD	0.33	0.2	0.26	0.26	0.19	0.17	0.23	0.26	0.21	0.18	0.18	0.38	0.28	0.22
GMR3	∅ deviation	1.17	1.17	0.77	1.25	1.14	1.29	1.43	1.37	1.09	1.26	1.04	1.01	0.96	1.19
	MAD	0.17	0.21	0.3	0.22	0.19	0.14	0.28	0.16	0.28	0.19	0.16	0.2	0.17	0.26

**Fig 6:**
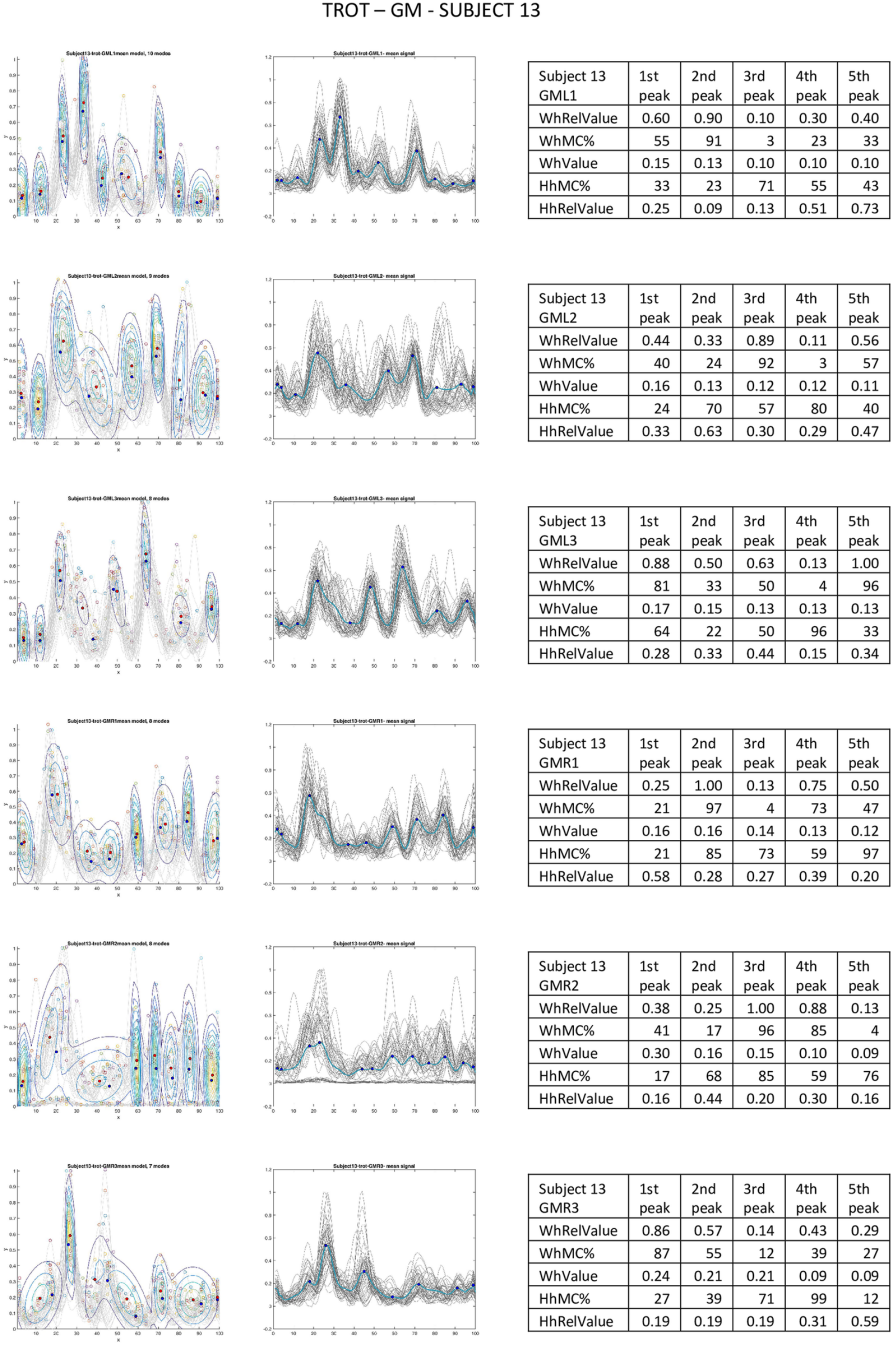
Trot of subject 13, m. gluteus medius. Leftmost column: GMMs per sensor. Middle column: curves (gray) of subject 13 per sensor location and mean curve (blue). Tables are based on the *i*th (1 ≤ *i* ≤ 5) highest peaks. WhRelValue: relative location (wrt total number of modes in GMM) of mode with *i*th highest contribution, *WhMC%*: frame number of mode with *i*th highest contribution within GMM (relative location wrt cycle length), *WhValue*: contribution value at WhMC%, *HhMC%*: frame number of *i*th highest peak within motion cycle (relative location wrt cycle length), *WhValue*: peak height at HhMC%.

**Fig 7:**
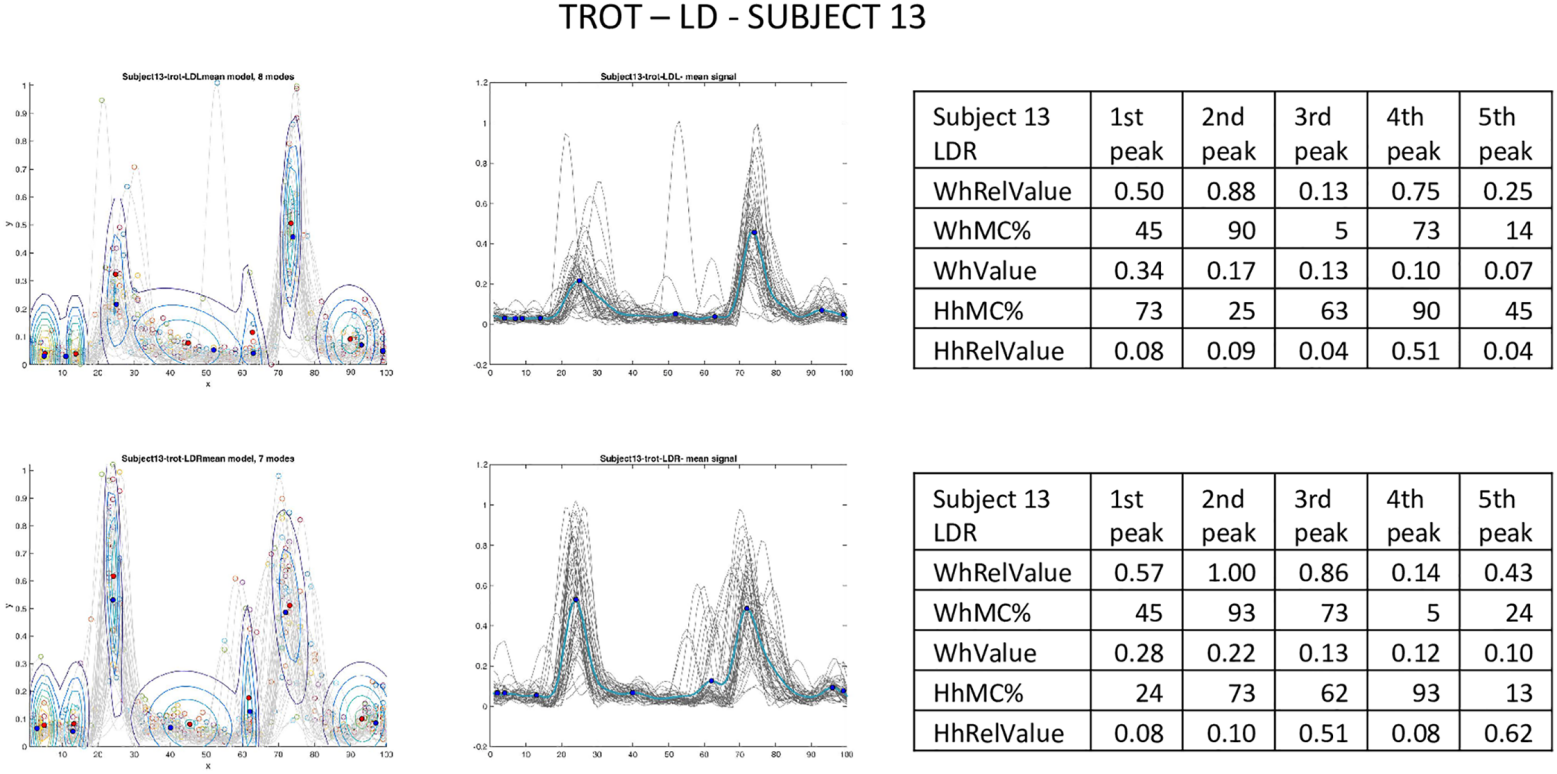
Trot of subject 13, m. longissimus dorsi. Leftmost column: GMMs per sensor. Middle column: curves (gray) of subject 13 per sensor location and mean curve (blue). Tables are based on the *i*th (1 ≤ *i* ≤ 5) highest peaks. WhRelValue: relative location (wrt total number of modes in GMM) of mode with *i*th highest contribution, *WhMC%*: frame number of mode with *i*th highest contribution within GMM (relative location wrt cycle length), *WhValue*: contribution value at WhMC%, *HhMC%*: frame number of *i*th highest peak within motion cycle (relative location wrt cycle length), *WhValue*: peak height at HhMC%.

**Fig 8:**
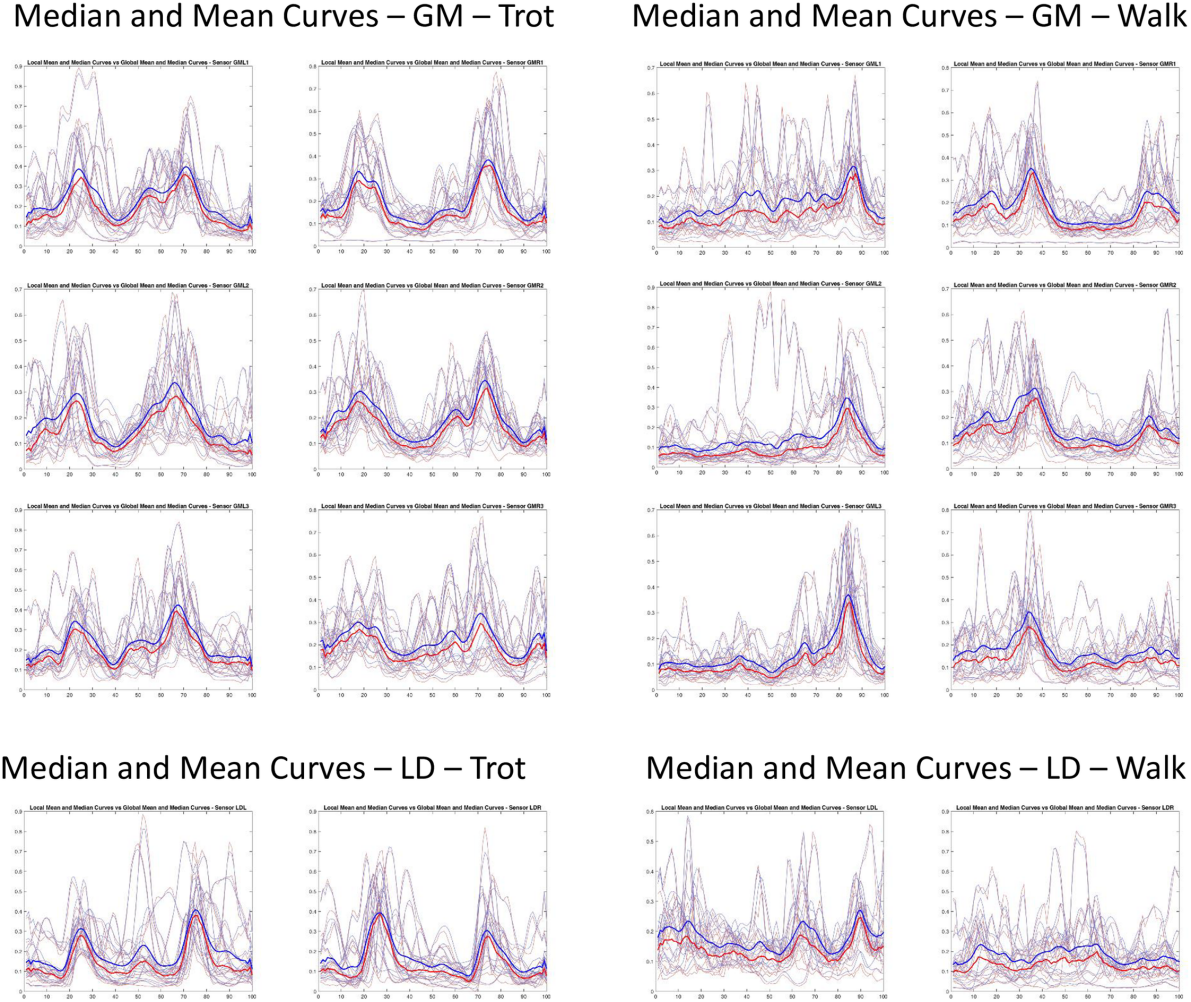
Activation pattern. Mean curves of all subjects per sensor (thin, blue), global mean curve per sensor (thick, blue) and median curves of all subjects per sensor (thin, red), global median curve per sensor (thick, red). It can be seen that there is a general two-peak activation pattern. Though this is observed on average data, individual cases differ vastly.

What can be seen from the numbers together with the visual presentations (Figs 6 and 7) that the activation patterns of individual subjects are consistent in a similar way as they represent subject 13 throughout their trials. That is, in each cycle of one gait the same type of activation pattern is found. The highest variability of subject 13 in trot was found in GM2 of all the sensors. This is consistent with what is seen in Fig 6. Note that the mean curve is presented instead of the median curve which may seem unrealistic because the mean curve does not represent an actual stream of data. However, when using the median curve, though there is a slight difference in height and a little less smoothness, but the difference is not as substantial as might be expected. See also Fig 8 for comparison.

The results of the intra-subject variability measures discussed in [11] are listed in Table 3. The values were computed by formulas 11 and 13, respectively median of Eq 14 which was given over time.

**Table 3:** Summary on intra-subject variability in trot. Evaluation by the measures discussed in [11].

Variability in Walk								
	LDL	GML1	GML2	GML3	LDR	GMR1	GMR2	GMR3
VR_median	0,75	0,64	0,60	0,55	0,74	0,57	0,62	0,66
CV_median	0,65	0,70	0,81	0,71	0,70	0,59	0,71	0,66
meanCV_median	0,59	0,68	0,70	0,64	0,64	0,56	0,59	0,63
CQV_median	0,39	0,40	0,41	0,40	0,38	0,34	0,38	0,38
Variability in Trot								
	LDL	GML1	GML2	GML3	LDR	GMR1	GMR2	GMR3
VR_median	0,44	0,49	0,54	0,52	0,42	0,49	0,53	0,61
CV_median	0,62	0,55	0,63	0,52	0,62	0,51	0,61	0,54
meanCV_median	0,60	0,53	0,57	0,53	0,58	0,50	0,60	0,54
CQV_median	0,36	0,35	0,36	0,34	0,35	0,32	0,35	0,37

#### Inter-Subject Variability of sEMG between Different Individuals

In addition to the consistency discussed in the section above, the focus was on finding the number of peaks which characterizes the signal recorded at each sensor location. By evaluation of the images comparing the individual mean curves of all subjects per sensor and by computing the global mean curve out of these. The reults of inter-subject variability computation can be found in Tables 4 and 5. There is roughly a two-peak structure visible in the data.

**Table 4:** Inter-Subject Deviations Trot.

Deviations from cycles to global mean curve per sensor															
	Subject number	1	2	3	4	5	6	7	8	9	10	11	12	13	14
LDL	∅ deviation	1.46	1.44	1.4	1.31	1.18	1.23	2.9	2.43	1.74	1.89	1.23	1.34	1.41	1.38
	MAD	0.16	0.23	0.32	0.22	0.18	0.12	0.37	0.29	0.41	0.25	0.23	0.24	0.14	0.21
GML1	∅ deviation	1.44	1.63	1.53	1.43	1.57	1.93	1.36	1.54	1.43	2.65	1.79	1.57	1.58	1.55
	MAD	0.26	0.3	0.3	0.2	0.11	0.25	0.12	0.27	0.22	0.21	0.33	0.27	0.17	0.24
GML2	∅ deviation	1.44	1.65	1.32	1.61	1.38	1.92	1.59	1.61	1.39	1.4	1.2	1.23	1.82	1.34
	MAD	0.23	0.39	0.16	0.22	0.29	0.25	0.35	0.29	0.35	0.22	0.18	0.23	0.47	0.23
GML3	∅ deviation	1.35	1.46	1.35	1.82	1.69	1.43	1.29	1.52	1.42	2.44	1.54	1.43	1.46	1.49
	MAD	0.18	0.22	0.19	0.24	0.18	0.12	0.19	0.19	0.24	0.26	0.18	0.27	0.17	0.14
LDR	∅ deviation	1.47	1.15	1.25	1.86	1.31	1.37	2.11	1.45	1.72	1.49	1.83	1.02	1.45	1.25
	MAD	0.17	0.16	0.2	0.37	0.2	0.36	0.29	0.16	0.35	0.23	0.24	0.18	0.29	0.23
GMR1	∅ deviation	1.56	1.39	1.4	2.05	1.19	1.43	1.24	1.58	1.51	1.41	1.33	1.92	1.38	1.21
	MAD	0.37	0.35	0.2	0.72	0.13	0.15	0.27	0.13	0.27	0.22	0.33	0.02	0.25	0.22
GMR2	∅ deviation	1.45	1.38	1.27	1.32	1.36	1.39	1.29	1.61	1.69	1.25	1.41	1.26	1.63	1.1
	MAD	0.26	0.33	0.2	0.33	0.29	0.25	0.18	0.38	0.37	0.22	0.22	0.31	0.27	0.2
GMR3	∅ deviation	1.77	1.55	1.68	1.45	1.81	1.56	1.72	1.67	1.71	1.65	1.7	1.45	1.46	1.58
	MAD	0.26	0.31	0.37	0.25	0.2	0.35	0.4	0.37	0.25	0.24	0.24	0.23	0.2	0.3

**Table 5:** Inter-Subject Deviations Walk.

Deviations from cycles to global mean curve per sensor															
	Subject number	1	2	3	4	5	6	7	8	9	10	11	12	13	14
LDL	∅ deviation	1.14	1.31	1.46	1.25	1.14	1.37	1.67	1.46	1.62	1.37	1.34	1.4	1.36	1.53
	MAD	0.24	0.44	0.27	0.25	0.22	0.27	0.21	0.26	0.27	0.3	0.3	0.19	0.19	0.34
GML1	∅ deviation	1.61	1.48	1.18	1.46	1.32	1.29	1.45	1.5	1.28	1.76	1.71	1.5	2.05	1.21
	MAD	0.36	0.44	0.17	0.21	0.16	0.22	0.17	0.21	0.16	0.43	0.31	0.18	0.3	0.13
GML2	∅ deviation	0.97	1.48	0.96	3.71	1.07	1.23	1.04	1.18	1.61	1.21	1.34	1.36	1.39	1.23
	MAD	0.23	0.35	0.17	0.32	0.18	0.26	0.09	0.29	0.22	0.29	0.28	0.11	0.26	0.16
GML3	∅ deviation	1.24	0.9	1.12	1.18	1.58	1.25	0.95	1.4	1.34	1.51	1.33	1.23	1.05	1.26
	MAD	0.3	0.21	0.29	0.23	0.18	0.18	0.13	0.25	0.28	0.3	0.15	0.26	0.16	0.25
LDR	∅ deviation	1.28	1.44	1.26	2.91	1.29	1.65	1.46	1.31	1.81	1.27	1.87	1.35	1.4	1.41
	MAD	0.39	0.34	0.21	0.49	0.28	0.21	0.2	0.2	0.48	0.28	0.17	0.16	0.38	0.31
GMR1	∅ deviation	1.3	1.84	1.34	1.93	1.37	1.64	1.66	1.25	1.47	1.4	1.36	1.72	1.45	1.28
	MAD	0.21	0.33	0.23	0.19	0.35	0.35	0.37	0.17	0.28	0.19	0.25	0.01	0.28	0.2
GMR2	∅ deviation	1.4	1.54	1.28	1.82	1.13	1.35	1.45	1.61	1.28	1.14	1.25	1.44	1.23	1.17
	MAD	0.16	0.32	0.25	0.33	0.24	0.25	0.19	0.38	0.25	0.16	0.23	0.17	0.18	0.15
GMR3	∅ deviation	1.32	1.82	1.31	1.51	1.48	1.53	1.78	1.84	1.29	1.46	1.34	1.15	1.33	1.38
	MAD	0.19	0.35	0.26	0.37	0.18	0.2	0.21	0.28	0.33	0.23	0.23	0.22	0.11	0.2

### Results of Mode Estimation

Biomechanics experts are interested in the peaks of EMG signals because they were assumed to be characteristic landmarks for the investigation of muscle activity. In the present study, activation of muscles is characterized by the location and height of peaks which also allows to relate phases of activation to phases of non-activation throughout motion cycles The created models are based on this information. However, they could as well have been based on the full set of discrete samples of the data curve given in our set of raw data.

One hypothesis based on the theory of GMM models indicates why it makes sense to rather model the peaks than the full set of points. The full point set is much more likely to produce overlapping components respectively modes with shared covariance. This will result in a mix of Gaussian modes in space and is also known to slow down the speed of convergence (cf Naim and Gildea [52]).

In practice, the difference between fitting the multi-modal model to the peaks of cycles as opposed to all available data can be evaluated resulting in the outcomes discussed in the following.

**Fig 9:**
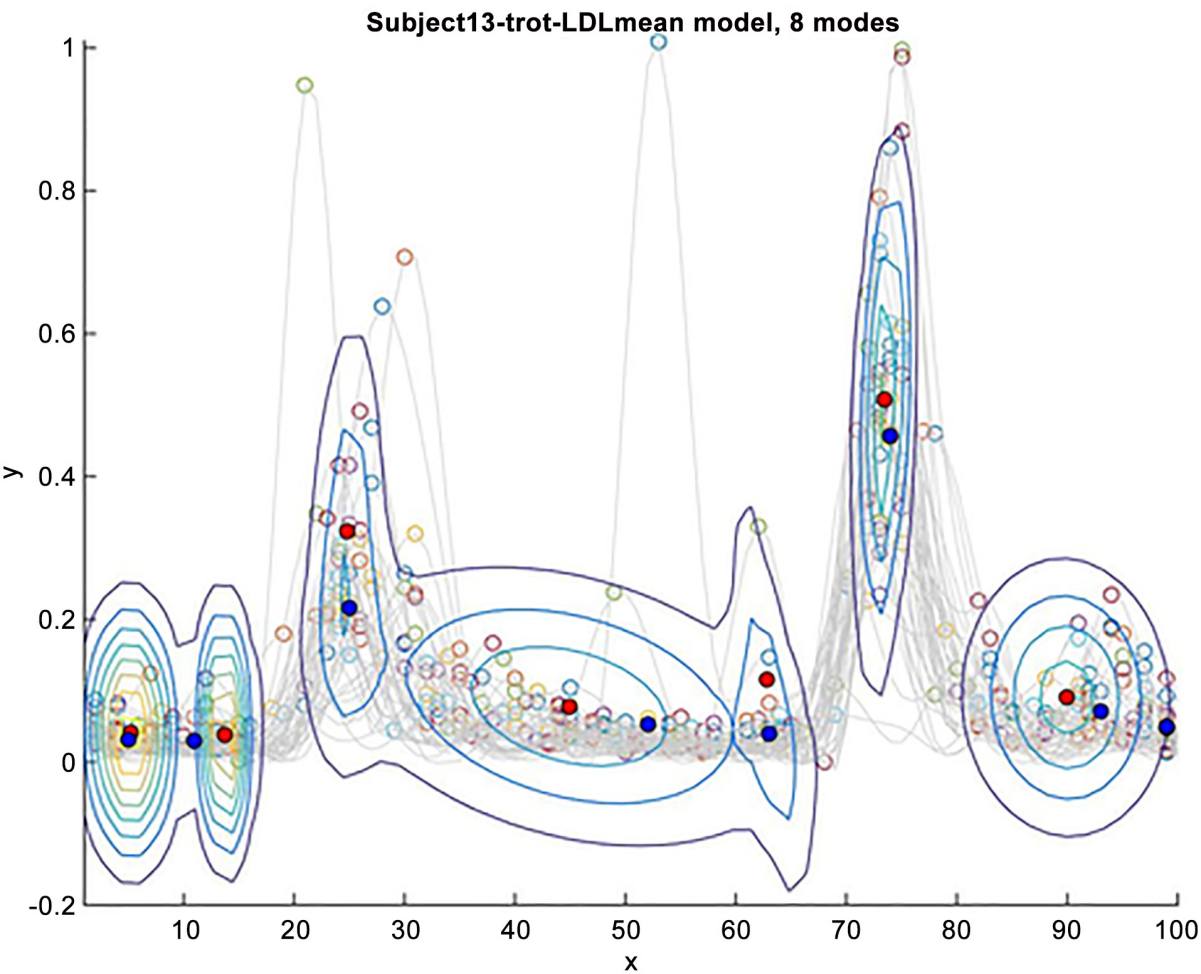
Peak-based model. Illustration of GMM model estimated by EM algorithm. Blue markers are peak positions of mean curve. Red markers are optimized means of GMM modes. Gray curves are data.

**Fig 10:**
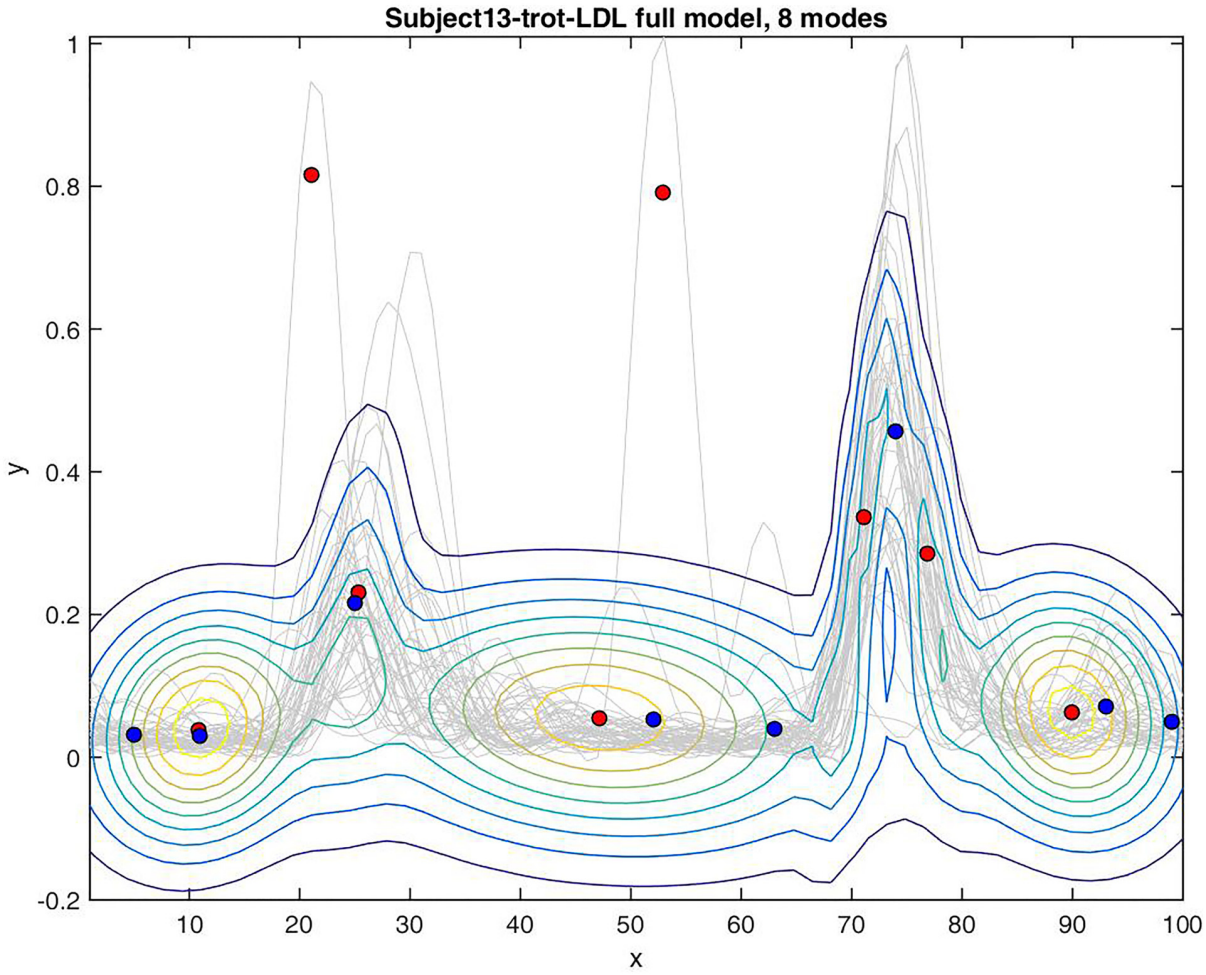
Model based on all data points. Illustration of GMM model estimated by EM algorithm. Blue markers are peak positions of mean curve. Red markers are optimized means of GMM modes. Gray curves are data.

A multi-modal bivariate Gaussian distribution was fitted to the full data set in the same way and with the same initialization as for the peak-based model. Since this produces the same number of Gaussian modes as for the peak-based model, this allows a straightforward comparison between the GMMs. As can be seen in the example image showing both scenarios (Figs 9 and 10 show an example for the left-side longissimus of subject 13), the peak-based models appear similar in quality to the models based on the complete signal. More precisely, the models based on the full data set overlap the models fitted to the peaks. In order to give a quantitative notion of this perceived similarity and in order to show that the peak-based models also suffice to characterize the signal, the similarity of the associated GMMs is computed.

The two sub-tables in Table 6 show the results of Cauchy-Schwarz distance computation per gait, sensor and individual of the group.

**Table 6:** Cauchy-Schwarz Distances of Multi-Modal Models Full vs. Peak-Based.

Cauchy-Schwarz Distances Trot																
Subject number	1	2	3	4	5	6	7	8	9	10	11	12	13	14	Median	MAD
LDL	0.27	0.15	0.16	0.13	0.18	0.13	0.32	0.23	0.53	0.28	0.2	0.18	0.13	0.12	0.18	0.05
GML1	0.31	0.27	0.22	0.2	0.71	0.33	0.21	0.16	0.71	0.33	0.54	0.67	0.26	0.73	0.32	0.11
GML2	0.24	0.26	0.2	0.19	0.55	0.13	0.25	0.17	0.1	0.22	0.18	0.09	0.21	0.1	0.19	0.05
GML3	0.18	0.23	0.13	0.23	0.27	0.15	0.17	0.25	0.3	0.4	0.63	0.47	0.3	0.21	0.24	0.06
LDR	0.25	0.2	0.15	0.7	0.14	0.16	0.32	0.12	0.11	0.71	0.18	0.21	0.13	0.13	0.17	0.04
GMR1	0.2	0.3	0.2	0.24	0.2	0.24	0.29	0.21	0.13	0.26	0.21	0.41	0.29	0.16	0.23	0.03
GMR2	0.09	0.15	0.17	0.2	0.34	0.19	0.22	0.16	0.17	0.21	0.22	0.24	0.18	0.1	0.18	0.03
GMR3	0.18	0.12	0.13	0.24	0.29	0.21	0.23	0.16	0.22	0.27	0.24	0.38	0.19	0.24	0.23	0.05
Median	0.22	0.22	0.17	0.21	0.28	0.18	0.24	0.17	0.2	0.28	0.22	0.31	0.19	0.14		
MAD	0.04	0.06	0.03	0.03	0.09	0.04	0.04	0.03	0.09	0.05	0.03	0.17	0.06	0.04		
Cauchy-Schwarz Distances Walk																
Subject number	1	2	3	4	5	6	7	8	9	10	11	12	13	14	Median	MAD
LDL	0.08	0.09	0.1	0.12	0.07	0.11	0.31	0.07	0.04	0.21	0.07	0.07	0.06	0.08	0.08	0.02
GML1	0.07	0.18	0.04	0.14	0.15	0.09	0.09	0.04	0.05	0.10	0.1	0.02	0.16	0.09	0.09	0.05
GML2	0.05	0.1	0.08	0.25	0.1	0.1	0.07	0.04	0.05	0.1	0.09	0.03	0.1	0.03	0.09	0.02
GML3	0.13	0.19	0.07	0.15	0.18	0.13	0.14	0.15	0.10	0.12	0.10	0.03	0.11	0.06	0.12	0.04
LDR	0.13	0.09	0.07	0.22	0.11	0.04	0.22	0.05	0.12	0.08	0.12	0.08	0.05	0.06	0.10	0.03
GMR1	0.06	0.17	0.1	0.2	0.1	0.05	0.14	0.07	0.12	0.08	0.03	0.05	0.16	0.04	0.09	0.03
GMR2	0.05	0.16	0.1	0.14	0.1	0.14	0.1	0.18	0.06	0.08	0.1	0.03	0.12	0.07	0.1	0.03
GMR3	0.07	0.28	0.05	0.11	0.15	0.11	0.03	0.12	0.11	0.08	0.07	0.09	0.09	0.06	0.09	0.02
Median	0.07	0.16	0.08	0.14	0.1	0.1	0.12	0.07	0.05	0.08	0.08	0.04	0.1	0.06		
MAD	0.01	0.04	0.02	0.02	0.02	0.01	0.04	0.03	0.01	0.009	0.01	0.01	0.01	0.02		

In sum, for all walk trials, the distances show that the two models are quite closely aligned with a median of 0.09 for all sensors. This shows that the models based on the maximally available number of peaks represent the signal sufficiently well. For the trot data, the median value was 0.22, however, the distances were generally higher than in walk. As can be seen in the table, the Cauchy-Schwarz distances also vary quite a lot between subjects as well as between different muscles. This is one observation that relates with the findings of intra-subject and inter-subject deviations discussed in the sections on variability.

In order to rate the distance values presented in Table 6 mean, comparative distance values are helpful. Therefore, a multi-modal model based on the same initialization was fitted to a stream of normally distributed noise. The Cauchy-Schwarz distances between this random multi-modal GMM and the peak-based GMM were computed. For trot, the distance values to the peak-based model were, on average, more than 4 times higher than the ones from the complete set of original data (displayed in Table 6) with a maximum factor of 9 times and a minimum factor of 2 times. For walk, The distance values to the peak-based model were, on average, more than 9 times higher than the ones from the complete set of original data (displayed in Table 6) with a maximum factor of 33 times and a minimum factor of 3 times.

Based on the discussion above, one important finding is that muscle activation signals of walk and trot can represented by GMMs of the collective peak patterns per cycle (one GMM accommodating all cycles of a set of trials capturing the same gait).

The specific results we obtained when doing so are discussed in the following. GMMs were fitted by the procedure described above. A most important observation is that the number of modes found by this method was always greater than one (minimum for trot: 5, minimum for walk: 6). This is not very common since, to date, one significant peak in sEMG data per cycle is expected [53]. This number also varied across different subjects and sensor positions (the ranges of this number of components for each subject is listed in Table 7.

**Table 7:** Maximum and Minimum Number of Modes Per Subject.

Max. and Min. in Trot														
Subject number	1	2	3	4	5	6	7	8	9	10	11	12	13	14
max # comps	10	9	9	9	10	9	11	9	10	11	9	11	10	8
min # comps	6	6	7	7	6	7	6	6	7	8	6	7	7	5
Max. and Min. in Walk														
Subject number	1	2	3	4	5	6	7	8	9	10	11	12	13	14
max # comps	11	12	10	13	10	11	12	11	11	10	10	11	12	11
min # comps	8	9	7	8	7	6	8	6	8	7	7	7	8	7

In particular it is clear that characterizing muscle activation by investigation of just one highest peak per cycle is not sufficient for either walk and trot and also for neither of the two different groups of muscles (longissimus dorsi and gluteus medius) investigated in our study. (cf Zsoldos et al. [53, 54]).

In order to find out what number of modes to focus on instead, two types of muscles were looked at in more detail.

#### Composite Peak Model of the M. Longissimus Dorsi

For the m. longissimus dorsi there is a strong indication of a two-peak pattern in trot. This can be seen especially well on average, i. e. by considering the mean curves per sensor over all individual trials and cycles (Fig 8). Note that the same pattern is observed in the median curves which are considered more well-suited for this type of experiment, e. g. by Carson et al. [55]. However, this two-peak pattern is not as apparent for some individuals. This is why it makes sense to distinguish groups of individuals for whom this is more apparent then for others (refer to the section on clustering).

Based on the assumption of a two-peak activation pattern in the sEMG signals of m. longissimus dorsi, the average distances were computed between the two highest peaks in the signals (separating the sensors bilaterally). Table 8 lists these average distances found between the two highest peaks for all subjects. The distance values fall into categories accordingly, this is quite consistent across the entire group of subjects.

**Table 8:** Distances between the two highest peaks found in the sEMG signal per cycle. Note that the distance is measured on a cycle, i. e. the “‘take the shortest route”’ is always taken even across the first respectively last frame (the distance between the first and last frame will be 1 instead of 99).

	WALK	WALK	WALK	WALK	TROT	TROT	TROT	TROT
	10–20	20–30	30–40	40–50	10–20	20–30	30–40	40–50
GML1	1	0	5	8	1	1	5	7
GML2	2	3	2	7	3	4	1	6
GML3	1	3	7	3	2	2	2	8
GMR1	0	2	5	7	1	2	3	8
GMR2	2	2	4	6	1	5	3	5
GMR3	1	3	2	8	0	3	3	8
LDL	1	2	6	5	1	4	4	5
LDR	1	0	6	7	2	2	2	2

Note that the two-peak assumption cannot be made for walk in the same way as for trot. However, the distances between the two highest peaks in the signals of the m. longissimus in walk are also quite consistent. All in all, this is a very interesting finding because this shows a significant difference between the activation patterns found in sEMG recordings of walk vs. trot.

#### Composite Peak Model of the M. Gluteus Medius

Longissimus and gluteus have different tasks and belong to different parts of the equine body. Therefore, it is not surprising that the activation patterns of the m. gluteus medius in individuals are entirely different compared to the ones of the longissimus dorsi. In fact, for individuals (cf Fig 6) the pattern can seem even chaotic.

Even more surprising is that for the m. gluteus medius there is also an indication of a two-peak pattern in trot. Again, this is a phenomenon which is observed well on the mean data, i. e. the mean curves per sensor over all individual trials and cycles (Fig 8), whereas for individuals the two-peak pattern is not as apparent. The classification of groups of individuals with different types of activation patterns is relevant here also.

On average, this suggests that there is a significant peak pattern in sEMG data of the m. gluteus medius which can be characterized by less than 5 peaks for different parts of this muscle.

Anyway, it can be seen that different parts of the m. gluteus respond differently to stimuli in terms of muscle activation. This is especially interesting in light of the results of Bruce [27] and Licka et al. [15, 16].

In the paper of Zsoldos et al. [53], the authors investigated just a single highest peak per cycle of signals acquired from the activation of different parts of the m. gluteus medius. This would be the equivalent of a single mode Gaussian model. However, as can be observed in Fig 6, the location of the highest peak per sensor can be scattered across the complete interval [1, …, 100]. This makes describing EMG signals by uni-modal Gaussians unreliable. Using the mean curve as initialization is a step towards more robust models and taking into account more than one peak allows for a more detailed analysis.

In summary, the two-peak structure may serve as a pointer towards a hypothesis about the relevant number of peaks to look out for in EMG signals of both types of muscle. However, differences in the signal structure between muscles, between individuals and also between different gaits can be expected. Using multi-modal GMMs is a more precise way of modeling muscle activation patterns of cycles in sEMG data.

### Results of Hierarchical Clustering

When highly heterogenous data is present, it makes sense to explore those by hierarchical clustering in the way introduced before. Employing the clustering step is an important step toward restructuring the data set. This will enable comparison of results of individual clusters with those of the complete group in order to identify candidates for the typical or average specimen and also to detect outliers in the groups. Hence, from this approach, new possibilities arise for analysis and exploration.

Physiologically, the m. longissimus dorsi and m gluteus medius serve for different tasks. So it makes sense to separate the two for a clustering.

#### Clustering of M. Longissimus Dorsi

Of the m. longissimus dorsi all information of corresponding sensors (‘LDL’ and ‘LDR’) was used. That is, for each of the two sensors

- The locations of the 5 highest peaks.
- The values of these peaks.
- The total number of modes for this sensor.

This sums up to a feature vector $f_{LD_{i}}\in R^{11}$ per sensor (1 ≤ *i* ≤ 2). Note that using 5 highest peaks per cycle is consistent with the upper limit for a number of peaks. This is due to fact that this was a minimum number of components for one of the subjects when fitting GMMs, Table 7, subject 14).

The features were used to compute a correlation matrix and from that, a dissimilarity matrix showing the pairwise Euclidean distances between any pair of feature values. According to these distances, agglomerative hierarchical clustering by using single linkage. Single link clustering with Euclidean distance measure was used. Single link means, in step three (see according section) when the distance of a new compound cluster to all other clusters is computed, the distance from the merged cluster to another cluster is equal to the shortest distance from any of its members to the outside cluster.

#### Clustering of M. Gluteus Medius

Of the m. gluteus medius all information of corresponding sensors (‘GML1-GML3’ and ‘GMR1-GMR3’) was used. That is, for each of the 6 sensors

- The locations of the 5 highest peaks.
- The values of these peaks.
- The total number of modes for this sensor.

This sums up to a feature vector $f_{GM_{j}}\in R^{54}$ per sensor (1 ≤ *j* ≤ 6). From there, the procedure was as described above.

The resulting clusterings for both groups of muscles are represented by cluster trees (Figs 4 and 3). Note that the images only show the cluster hierarchies from the level at which there were 4 different clusters to the top. Since agglomerative clustering is a bottom-up approach, this leaves out the first stages at which each individual was in its own cluster.

## Conclusions

New modeling techniques were introduced for the analysis of quadrupedal sEMG processing. In the present study, hierarchical clustering techniques were combined with a state of the art pre-processing pipeline, thus detecting patterns in sEMG data. This means a step towards the analysis of such data sets. Since there is a yet lack of standardization of animal sEMG signals, this work tested a variety of methods toward the identification of coherent patterns in such data sets. With the help of these tools, experts in the field will be able to acquire new insight in the structure of sEMG readings on different levels. Establishing normalization techniques like the one available for research in human biomechanics [12] would even further improve this analysis of animal data. If processing sEMG recordings of animal muscle activation is to face the same challenges as processing human sEMG, there should be a summary what information can be gained from animal sEMG data, similarly what is provided for research in humans [1].

By means of the methods introduced in this paper, composite peak models were identified per individual for all 14 horses in the study. This composite peak model represents the general activation pattern per sensor location.

All in all, the data set used in the current study displayed higher variability on an inter-subject level than expected. Even though the group of horses was homogenous (all were mares, all were from the same breed, all were close in age), the activation patterns differed between individuals. Granata et al. [50] documented phasic EMG waveform patterns variability in walking children and interpreted their level controversially. This level of inter-subject variability together with the fact that coherent muscle activation patterns were found on an intra-subject level, is a very interesting finding. Differences in muscle activation that are found across individuals can now be investigated further and a partition all the complete group with respect to their functional characteristics is possible. This may even bring a new perspective into future research. Namely, whether it is more important to analyze individuality in heterogenous data of otherwise consistent groups of subjects or is it more desirable to focus on analyzing the common features within such groups. While this question has not been answered in the field of animal electromyography, questions have risen about how heterogenous muscle activation estimates can be and how to deal with this (cf. Staudenmann et al. [56]). Both objectives, investigation of individuality and analysis of general features, could be of equal importance. In fact, a hierarchical exploration of data brings interesting opportunities to pursue both. A new way of doing doing this using hierarchical clustering was outlined in the present study.

The current study will contribute to progress in the field of animal muscle mechanics by introducing new techniques to explore sEMG signals of quadrupeds in terms of individual and common aspects. It is especially important to further standardize this type of research, thus closing the gap between animal and human electromyography.

With the help of the methods presented in this paper the differences in muscle activation between different gaits were found on both general and individual level. Such differences as might be expected also occurred between the two different groups of muscles. Especially in walk, the differences between m.longissimus dorsi and m. gluteus medius were clearly visible.

By applying three different sensors to the m. gluteus medius muscles of both sides, we have demonstrated that, in each individual, there are strong indications of a large difference, while on group average level, the difference was less apparent but still present. While it was important to note this divergence in general, it will be an interesting topic for future research to identify in what way the peaks observed at different locations of the same muscle will differ. This indicates the role of muscle specificity in the functional use of body parts, e. g. for locomotion. Differences in muscle specificity may answer the question of why there are differences and overlaps between the muscle activity patterns across individuals of the same species and breed. Each of the muscles investigated in the study has multiple functions. For example, the m. longissimus dorsi has the function of extending the back but also of stabilizing the back, in m. gluteus medius the functions of flexing the leg but also of protracting the limb forward to initiate a step. There are always individual solutions for different subjects, such as some will flex one or more legs more than the others will. This is a likely cause of the differences observed in different individuals. Also, it is quite a complex source of individual differences in patterns observed in the same bilateral muscle or even in different sections of one muscle.

### Outlook

Employing statistical methods for modeling of sEMG signals has shown that these signals are sufficiently represented by a maximally available number of peaks. It would be interesting to see if creating more compact GMMs is possible. We propose merging adjacent modes of one GMM based on their Kullback-Leibler distance and or the divergence in direction of principal axes. The Kullback-Leibler divergence could well be computed between uni-modal Gaussians. This could yield a more accurate classification of individuals by allowing a controlled overlap of peaks.

In our GMM fitting approach, estimation of modes was initialized by the maximally available number of peaks of a mean curve per sensor and was optimized based on that number. Doing this more automatically by optimizing for different error functions could help. The works of Melnykov and Melnykov [44] propose a strategy initializing mean vectors by choosing points with higher concentrations of neighbors and using a truncated normal distribution for the preliminary estimation.

For future work, it has to be noted that the mean activation has a risk of being too prone to distortion by high inter-individual variability values. As a remedy for this, after classification of individuals by our clustering method, the mean and median curves could again be compared for each cluster. Doing this at different stages of our clustering hierarchy would be a potentially very interesting step towards diagnosis of differences between the clustered groups of individuals. This could eventually help investigating differences of activation in cohorts of population and relating these to meta-level information on these cohorts. As such a large new study was outwith the remit of the present study, we are considering an additional study into this for a follow up paper.

Since the number of subjects in our group was too small to allow for a larger number of clusters in the clustering step, one would ideally start out with a larger group of individuals to create larger subgroups of subjects. Hypothetically, this could be extended to capturing a larger data pool allowing for comparison of sEMG patterns between species. Eventually, the task would be extended to investigating muscle activation patterns during other gaits and movements in order to show how our results transfer to other scenarios.

Taking into account the anatomical differences such as found at an intra-muscular level could help explain pattern differences observed between different parts of the same muscle. Small-scale differences in the nerve supply or in the distribution of nerve branches could have significant influence on activation timing. Since nerve branches are spread, branches located closer to the main branch could cause muscle parts to activate earlier with respect to other parts. For related ideas in human research cf Watanabe et al. [57].

When evaluating the approach of segmenting the data into cycles of gaits the question arises whether splitting sequences at this point causes a problem with redundant modes. Though locating ground contacts of a specific hoof has been a simple task, this does not answer the question if this may have split data in a way that one group of peaks will be separated such that some of its members are in the beginning of the cycles and some will be located at the end. In the worst case, this would lead to one additional mode (at the end) where there is actually just one (in the beginning). Splitting cycles at a different point would only shift the same problem to a different stage of the gait. Creating an overlap of semi-cycles at the beginning and at the end of each cycle could help alleviate this. A *panoramic* view of frame interval [*f*_−50_, …, *f*_150_] of each cycle could make it easier to exclude errors by inept segmentation. All in all, new innovative ways of looking movement pattern would be beneficial in order to get away from the classical gait cycle analysis methods towards gaining new insight in muscle functions of the described type.

## Supporting Information

S1 File Supporting information file containing all data recorded of 14 test subjects in trot. (ZIP)

S2 File Supporting information file containing all data recorded of 14 test subjects in walk. (ZIP)
